# Galectin-3 Binding Protein, Depression, and Younger Age Were Independently Associated With Alexithymia in Adult Patients With Type 1 Diabetes

**DOI:** 10.3389/fpsyt.2021.672931

**Published:** 2021-05-11

**Authors:** Eva O. Melin, Ralph Svensson, Jonatan Dereke, Magnus Hillman

**Affiliations:** ^1^Diabetes Research Laboratory, Department of Clinical Sciences, Faculty of Medicine, Lund University, Lund, Sweden; ^2^Region Kronoberg, Department of Research and Development, Växjö, Sweden; ^3^Department of Psychology, Linnaeus University, Växjö, Sweden

**Keywords:** alexithymia, depression, galectin-3, galectin-3 binding protein, glycemic control, obesity, sCD163, type 1 diabetes

## Abstract

**Aims:** Alexithymia has been linked to cardiovascular disease. The aim was to explore whether the immuno-inflammatory variables galectin-3 binding protein (Gal3BP), soluble (s)CD163 and galectin-3 were independently associated with alexithymia, while controlling for known risk factors for cardiovascular disease, such as depression, anxiety, impaired glycemic control, obesity, smoking, and physical inactivity in patients with type 1 diabetes (T1D).

**Methods:** Cross-sectional design. The participants were consecutively recruited from one diabetes out-patient clinic. Alexithymia, depression and anxiety were assessed by self-report instruments. Blood samples, anthropometrics, and blood pressure were collected, supplemented with data from electronic health records. High Gal3BP was defined as ≥3.3 μg/ml, high sCD163 as ≥0.6 μg/ml, high galectin-3 as ≥2.6 ng/ml, impaired glycemic control as HbA1c >70 mmol/mol (>8.6%) and abdominal obesity as waist circumference ≥ 1.02 m for men and ≥ 0.88 m for women.

**Results:** Two hundred and ninety two patients participated (men 56%, aged 18–59 years, alexithymia prevalence 15%). Patients with alexithymia had higher prevalence of depression (34 vs. 6%, *p* < 0.001), anxiety (61 vs. 30%, *p* < 0.001), high Gal3BP levels (39 vs. 17%, *p* = 0.004), high HbA1c levels (46 vs. 24%, *p* = 0.006), and abdominal obesity (29 vs. 15%, *p* = 0.045). Depression [adjusted odds ratio (AOR) 6.5*, p* < 0.001], high Gal3BP levels (AOR 2.4, *p* = 0.035), and age (AOR 0.96, *p* = 0.027) were independently associated with alexithymia. Abdominal obesity (AOR 4.0, *p* < 0.001), high Gal3BP levels (AOR 2.8, *p* = 0.002), and depression (AOR 2.9, *p* = 0.014) were associated with high HbA1c. Abdominal obesity and anxiety were associated [Crude odds ratio (COR) 2.4, *p* = 0.006].

**Conclusions:** T1D patients with alexithymia had higher prevalence of high Gal3BP levels, depression, impaired glycemic control, anxiety, and abdominal obesity, which are known risk factors for cardiovascular disease. Only high Gal3BP levels, depression, and younger age were independently associated with alexithymia in adult patients with T1D.

## Introduction

Alexithymia literally denotes “no words for feelings.” Alexithymia is a state or personality trait characterized by limited ability to identify and describe feelings, and to distinguish between sensations in the body caused by emotional arousal and sensations of other origins (1–3). An externally oriented cognitive style and constricted imaginal processes are other features of alexithymia (1–3). Alexithymia is also characterized by inflexible emotion regulation with inadequate emotional reactivity, prolonged emotional states and failed habituation (4). Emotional neglect, physical or sexual abuse during childhood, as well as neuropsychiatric and genetic disturbances may cause alexithymia (5–7).

Alexithymia has been linked to increased cardiovascular disease and mortality (8, 9), type1 diabetes (T1D) (10), obesity (11, 12), higher blood pressure and dyslipidaemia (13), depression (9, 14–16), and anxiety (17, 18). Increased baseline levels of sympathetic activity and disturbances of the hypothalamo-pituitary-adrenal (HPA) axis have been demonstrated in persons with alexithymia (2, 3). Both the increased chronic sympathetic arousal and the disturbances of the HPA axis make persons with alexithymia highly vulnerable to chronic stress conditions (2, 3). It has been suggested that alexithymia impacts the function of the human immune system both through the sympathetic overreactivity and the activation of the HPA axis (2, 3). Disturbances of the immune system, involving monocytes and macrophages, contribute to the development of atherosclerosis and cardiovascular disease (19–25). Galectin-3 binding protein (Gal3BP) is a macrophage scavenger receptor which, while activated, induces a number of pro-inflammatory cytokines (21). Gal3BP, which is also known as Mac-2BP, LGALS3BP or 90K, is part of the innate immune system (26). Increased plasma levels of Gal3BP have been linked to metabolic disturbances (27), to recurrent angina/myocardial infarction (22), and to cardiovascular and all-cause mortality (21). CD163 is a macrophage and monocyte expressed scavenger receptor (28). After ectodomain shedding, the extracellular portion of CD163 circulates in blood as a soluble protein (sCD163) (29). The sCD163 levels increase during macrophage activation (29). Increased sCD163 levels have been linked to acute coronary syndrome (23). Galectin-3 is a beta-galactoside-binding lectin involved in several inflammatory processes (30). Galectin-3 has been linked to depression (31, 32), heart failure (24), coronary artery disease (25), and all-cause mortality (22).

Other factors previously linked to cardiovascular disease and mortality are metabolic disturbances (13, 33, 34), T1D (35), physical inactivity (36), smoking (37), depression (38), and anxiety (39). Depression has also been linked to several inflammatory, metabolic and HPA-axis disturbances (15, 31, 32, 40–44), which is also the case for obesity, smoking and physical inactivity (15, 36, 37, 42, 45). T1D is an auto-immune disorder characterized by insulin-deficiency (46), which is accompanied by several metabolic disturbances (33, 34).

As alexithymia previously has been linked to cardiovascular disease and mortality (8, 9), we wanted to explore whether selected known risk factors for cardiovascular disease and mortality were directly or indirectly linked with alexithymia in patients with T1D. The main aim was to explore whether the immuno-inflammatory variables Gal3BP, sCD163, and galectin-3 were independently associated with alexithymia controlling for depression, anxiety, metabolic and life style factors, medication, and cardiovascular complications.

## Materials and Methods

### Participants and Study Design

The study has a cross sectional design and included 292 (69%) patients out of 424 eligible patients with T1D (15). Inclusion criteria were T1D of ≥1-year duration, in patients 18–59 years of age. Exclusion criteria were pregnancy, severe somatic and psychiatric disorders such as cancer, hepatic failure, end-stage renal disease, Cushing's disease, severe autoimmune disorders, psychotic disorders such as schizophrenia, bipolar disorder, severe personality disorders, severe substance abuse, cognitive deficiency (due to stroke, dementia, or intellectual disability), or inadequate knowledge of the Swedish language. No patients used antipsychotic drugs (15). The patients were consecutively recruited during a 9-month period, from 25 March 2009 to 28 December 2009, from the largest out of two hospital diabetes outpatient clinics in Region Kronoberg, Sweden. The catchment population was 125,000. Self-report questionnaires were used to assess alexithymia, depression and anxiety. Blood samples, anthropometrics, and blood pressure were collected, supplemented with data from electronic health records (12, 15, 31, 40–42, 47–49). The study was performed in accordance with the Declaration of Helsinki, and was approved by the Regional Ethical Review Board of Linköping University, Linköping (Registration no. M120-07, T89-08). All participants provided written informed consent.

### Definitions of Alexithymia, Depression, and Anxiety

Alexithymia was assessed by Toronto Alexithymia Scale-20 items (TAS-20) and alexithymia was defined as ≥ 61 points (3, 8, 9, 11, 12, 14, 15, 17, 18, 50–52). TAS-20 has a three-factor structure with three subscales, “Difficulty Identifying Feelings” (DIF), “Difficulty Describing Feelings” (DDF) and “Externally Oriented Thinking” (EOT) (50, 51). The alexithymia characteristic of constricted emotional processes is not elucidated by TAS-20.

Depression and anxiety were assessed by Hospital Anxiety and Depression Scale (HADS) Depression was defined as HADS—the depression subscale (HADS—D) ≥8 points and anxiety as HADS—the anxiety subscale (HADS—A) ≥8 points (15, 31, 42, 53).

### Definitions of Abdominal and General Obesity, Severe Hypoglycaemia Episodes, and Cardiovascular Complications

Abdominal obesity was defined as waist circumference (WC) ≥1.02 m for men and as WC ≥0.88 m for women, and general obesity was defined as BMI ≥30 kg/m^2^ for both sexes. A severe episode of hypoglycaemia was defined as hypoglycaemia to such a degree that the patient needed help from another person. Severe hypoglycaemia episodes occurring during a period of 6 months prior to inclusion were included. Cardiovascular complications were defined as cardiac failure, ischemic heart disease, stroke, or transient ischemic attack (TIA) (15).

### Smoking and Physical Activity

Smokers were defined as having smoked any amount of tobacco during the last year (15). There were four categories of physical activity including at least 30 min of moderate activity: less than once a week, 1–2 times a week, 3–5 times a week, and daily.

### Blood Pressure, Antihypertensive Drugs, and Indications for Treatment of Hypertension

Blood pressure was measured in the sitting position by a nurse. The antihypertensive drugs which were used by the patients were previously described (41). The use of antihypertensive drugs was dichotomized into users and non-users (41). Indications for antihypertensive drugs were systolic blood pressure >130 mm Hg and/or diastolic blood pressure >80 mm Hg (41).

### Serum-Lipids, Lipid-Lowering Drugs, and Indications for Treatment of Hyperlipidemia

Serum-lipids were collected after an overnight fast. Lipid lowering drugs were HMG CoA-reductase inhibitors, and the use of lipid lowering drugs was dichotomized into users and non-users (41). Indications for lipid lowering drugs were total cholesterol (TC) >4.5 mmol/l (>1.74 mg/dl) and/or LDL-cholesterol >2.5 mmol/l (>97 mg/dl) (41).

### Insulin

Patients used either multiple daily insulin injections (MDII) or continuous subcutaneous insulin infusion (CSII).

### Antidepressants

The antidepressants which were used by the patients were previously described (41). The use of antidepressants was dichotomized into users and non-users.

### Biochemical Analyses

Plasma levels of Gal3BP, sCD163 and galectin-3 were measured using commercially available DuoSet enzyme linked immune-sorbent assay (ELISA) kits (R&D Systems, Minneapolis, Minnesota, USA) and optimized for human plasma. The analyses were run according to the manufacturer's instructions (31, 47, 48). For the Gal3BP, sCD163, and galectin-3 analyses the samples were diluted 1:4,000, 1:2, and 1:200, and the intra-assay coefficients were 3.9, 4.3, and 2%, respectively. All samples were run as duplicates. High levels of Gal3BP were defined as ≥3.3 μg/ml (≥80th percentile) (47), high levels of sCD163 as ≥0.6 μg/ml (≥80th percentile) (48), and high levels of galectin-3 were defined as ≥2.6 ng/ml (≥85th percentile) (31).

HbA1c [mmol/mol (%)], serum-lipids (mmol/l), and creatinine (μmol/l) were collected. High levels of HbA1c were defined as > 70 mmol/mol (> 8.6%). Methods used for the biochemical analyses and the intra-assay coefficients of variation for each of these variables were previously described (41).

### Statistical Analysis

Analysis of data distribution using histograms revealed that age, diabetes duration, Gal3BP, sCD163, galectin-3, triglycerides, systolic, and diastolic BP, were not normally distributed. Data were presented as median [quartile (q)_1_, q_3_; min-max], and analyses were performed with Mann-Whitney *U* test. Fisher's Exact Test (two-tailed) and Linear-by-linear association were used to analyse categorical data, and data were presented as N (%).

Medians and prevalence rates for the variables included in the study were compared between patients with and without alexithymia. Variables where these comparisons showed *P* < 0.10, and sex, age, and cardiovascular complications independent of *P*-values, were included in the further analyses. Crude odds ratios (CORs) for these variables were calculated with alexithymia as dependent variable. Variables with *P* < 0.10 for the CORs, and age and sex independent of *P*-values, were entered into multiple logistic regression analysis (Backward: Wald) with alexithymia as dependent variable in three separate models, and with HbA1c >70 mmol/mol as dependent variable in one model, and with cardiovascular complications as dependent variable in one model (54), The Hosmer and Lemeshow test for goodness-of-fit and Nagelkerke R^2^ were used to evaluate each multiple logistic regression analysis model. ROC analyses were performed. After log transformation (Lg10) of Gal3BP, multiple linear regression analysis (Backward) was performed with Gal3BP (Lg10) as dependent variable and with alexithymia, depression, and anxiety as independent variables. Bivariate and partial correlation analyses were performed. Pearson correlation coefficients, collinearity tolerance coefficients and variance inflation factor (VIF) coefficients were calculated. Confidence intervals (CIs) of 95% were used. *P* < 0.05 was considered statistically significant. SPSS® version 25 (IBM, Chicago, Il, USA) was used.

## Results

Baseline characteristics were compared between 44 (15%) T1D patients with and 248 (85%) T1D patients without alexithymia in Table 1. Comparisons showed that the patients with alexithymia compared to the patients without alexithymia had 5.7 times higher prevalence of depression (34 vs. 6%, *p* < 0.001), twice as high prevalence of anxiety (61 vs. 30%, *p* < 0.001), 1.9 times higher prevalence of abdominal obesity (29 vs. 15%, *p* = 0.045), and 3.2 times higher prevalence of combined anxiety and abdominal obesity (21 vs. 6.5%).

**Table 1:** Baseline characteristics compared between 44 T1D patients with and 248 T1D patients without alexithymia.

		**All patients^a^**	**Alexithymia**		
			**Yes**	**No**	***P-*value^b^**
*N*		292	44	248	
Age (years)		(18–59)	38 (27, 51)	42 (32, 51)	0.26
Diabetes duration (years)		(1–55)	18 (9, 24)	20 (11, 31)	0.087
Sex	Women	130 (44)	24 (54)	142 (57)	0.19^c^
	Men	162 (56)	20 (46)	106 (43)	
Depression		30 (10)	15 (34)	15 (6)	<0.001^c^
Anxiety		101 (35)	27 (61)	74 (30)	<0.001^c^
Abdominal obesity^d^		49 (17)	12 (29)	37 (15)	0.045^c^
Anxiety and abdominal obesity combinations	Anxiety/abdominal obesity combined	25 (9)	9 (21)	16 (6.5)	0.001^c^
	Anxiety only	72 (25)	16 (38)	56 (23)	
	Abdominal obesity only	24 (8)	3 (7)	21 (8.5)	
	No anxiety/no abdominal obesity	165 (58)	14 (33)	151 (62)	
General obesity^e^		36 (12)	9 (21)	27 (11)	0.079^c^
Systolic blood pressure (mm Hg)		(90–160)	120 (110, 130)	120 (110, 130)	0.75
Diastolic blood pressure (mm Hg)		(55–100)	70 (70, 78)	70 (70, 75)	0.59
Hypoglycemia (severe episodes)		13 (4)	2 (4)	11 (4)	<0.99^c^
Smoking^f^		28 (10)	6 (15)	22 (9)	0.26^c^
Physical inactivity^g^	daily	99 (36)	14 (35)	85 (36)	0.84^h^
	3–5 times/week	86 (31)	16 (40)	70 (30)	
	1–2 times/week	59 (22)	4 (10)	55 (23)	
	<1 time/week	31 (11)	6 (15)	25 (11)	
Continuous subcutaneous insulin infusion		27 (9)	1 (2)	26 (10)	0.095^c^
Antidepressants		23 (8)	6 (14)	17 (7)	0.13^c^
Lipid lowering drugs		135 (46)	21 (48)	114 (46)	0.87^c^
Antihypertensive drugs		97 (33)	15 (34)	82 (33)	>0.99^c^
Cardiovascular complications		10 (3)	3 (7)	7 (3)	0.18^c^

a *Data are presented as (min-max) or N (%)*.

b *Mann-Whitney U test unless indicated, data are presented as median (q_1_, q_3_)*.

c *Fisher's Exact test. Missing values (N)*:

d *6*;

e *2*;

f,g *17*.

h *Linear—by linear association*.

Results from the biochemical analyses are presented in Table 2. Comparisons showed that the patients with alexithymia compared to the patients without alexithymia had 2.3 times higher prevalence of high Gal3BP (39 vs. 17%, *p* = 0.004), 1.7 times higher prevalence of high sCD163 (30 vs. 18%, *p* = 0.095), and 1.9 times higher prevalence of high HbA1c (> 70 mmol/mol) (46 vs. 24%, *p* = 0.006). The patients with alexithymia did not have higher prevalence of high galectin-3, but had higher median galectin-3 levels (*p* = 0.005).

**Table 2:** Results from biochemical analyses compared between 44 T1D patients with and 248 T1D patients without alexithymia.

		**All patients^a^**	**Alexithymia**		
			**Yes**	**No**	***P-*value^b^**
*N*		292	44	248	
Gal3BP (μg/ml)^c^		(0.8–8.9)	2.8 (2.3, 4.2)	2.2 (1.7, 2.9)	<0.001
High Gal3BP (≥3.3 μg/ml)^c^		59 (21)	17 (39)	42 (17)	0.004^d^
sCD163 (μg/ml)^e^		(0.2–1.9)	0.5 (0.4, 0.6)	0.4 (0.3, 0.5)	0.084
High sCD163 (≥0.6 μg/ml)^e^		57 (20)	13 (30)	44 (18)	0.095^d^
Galectin-3 (ng/ml)^f^		(0.001–100.0)	1.4 (0.8, 2.2)	0.9 (0.5, 1.6)	0.005
High Galectin-3 (≥2.6 ng/ml)^f^		42 (15)	9 (20)	33 (14)	0.25^d^
HbA1c (mmol/mol)		(25–110)	68 (56, 78)	62 (54, 70)	0.007
(%)		(4.4–12.2)	8.4 (7.3, 9.3)	7.8 (7.1, 8.5)	
HbA1c > 70 mmol/mol (> 8.6%)		80 (27)	20 (46)	60 (24)	0.006^d^
Total cholesterol (mmol/l)		(2.1–10.9)	4.6 (4.0, 5.4)	4.6 (4.1, 5.2)	0.87
LDL-cholesterol (mmol/l)		(0.6–8.3)	2.8 (2.4, 3.6)	2.8 (2.4, 3.3)	0.58
Triglycerides (mmol/l)		(0.1–5.9)	0.9 (0.6, 1.8)	0.9 (0.7, 1.2)	0.23
HDL-cholesterol (mmol/l)		(0.3–2.7)	1.4 (1.2, 1.7)	1.5 (1.3, 1.8)	0.060
S-Creatinine (μmol/l)^g^		(28–182)	70 (64, 76)	70 (61, 78)	0.97

a *Data are presented as (min-max)*.

b *Mann-Whitney U test unless otherwise indicated, data are presented as median (q_1_, q_3_)*.

*Missing value*:

c *7*.

d *Fisher's Exact Test. Missing values*:

e *5*;

f *9*,

g *13*.

Associations with alexithymia are presented in Table 3 for three models. Model 1: depression [adjusted odds ratio (AOR) 6.5*, p* < 0.001], age (AOR 0.96, *p* = 0.027), and high Gal3BP levels (≥ 3.3 μg/ml) (AOR 2.4, *p* = 0.035), were associated with alexithymia. Model 2: depression (AOR 6.6, *p* < 0.001), combined anxiety and abdominal obesity (AOR 4.9, *p* = 0.004), age (AOR 0.96, *p* = 0.028), and high Gal3BP levels (≥ 3.3 μg/ml) (AOR 2.4, *p* = 0.039), were associated alexithymia. Model 3: depression (AOR 5.8, *p* = 0.001), Gal3BP (per μg/ml) (AOR 1.6, *p* = 0.002), combined anxiety and abdominal obesity (AOR 5.1, *p* = 0.004), and age (AOR 0.96, *p* = 0.034), were independently associated with alexithymia.

**Table 3:** Associations with alexithymia in 292 T1D patients explored for three different models.

	**Alexithymia**							
			**Model 1**		**Model 2**		**Model 3**	
	**COR (95% CI)**	***P*-value**	**AOR (95% CI)**	***P*-value^a^**	**AOR (95% CI)**	***P*-value^b^**	**AOR (95% CI)**	***P*-value^c^**
Age (per year)	0.98 (0.96–1.01)	0.21	0.96 (0.93–1.00)	0.027	0.96 (0.93–1.00)	0.028	0.96 (0.93–1.00)	0.034
Diabetes duration (per year)	0.98 (0.95–1.003)	0.079	0.98 (0.95–1.02)	0.42	0.98 (0.95–1.02)	0.42	0.98 (0.95–1.02)	0.43
Sex (women)	1.6 (0.8–3.1)	0.15	0.8 (0.4–2.0)	0.69	0.8 (0.4–2.0)	0.69	0.7 (0.3–1.6)	0.41
Depression	8.0 (3.6–18.1)	<0.001	6.5 (2.4–17.4)	<0.001	6.6 (2.4–17.9)	<0.001	5.8 (2.1–16.1)	0.001
Anxiety	3.7 (1.9–7.3)	<0.001	2.0 (0.9–4.5)	0.094	–	–		–
Abdominal obesity	2.2 (1.1–4.8)	0.037	2.4 (1.0–5.8)	0.050	–	–	–	–
Anxiety/abdominal obesity combinations	–	–	–	–	–		–	–
Anxiety and abdominal obesity combined	6.1 (2.3–16.2)	<0.001	–	–	4.9 (1.6–14.6)	0.004	5.1 (1.7–15.4)	0.004
Anxiety only	3.1 (1.4–6.7)	0.005	–	–	1.9 (0.7–4.8)	0.18	2.2 (0.9–5.9)	0.102
Abdominal obesity only	1.5 (0.4–5.8)	0.52	–	–	2.2 (0.5–8.7)	0.28	1.8 (0.4–7.6)	0.39
No anxiety/no abdominal obesity	1		–	–	1	–	1	–
Gal3BP (per μg/ml)	1.6 (1.3–2.1)	<0.001	–	–	–	–	1.6 (1.2–2.1)	0.002
High Gal3BP (≥ 3.3 μg/ml)	3.0 (1.5–6.0)	0.002	2.4 (1.1–5.4)	0.035	2.4 (1.0–5.4)	0.039	–	–
sCD163 (per μg/ml)	2.2 (0.6–7.9)	0.23	–	–	–		–	–
High sCD163 (≥0.6 μg/ml)	2.0 (1.0–4.1)	0.068	1.4 (0.6–3.4)	0.43	1.4 (0.6–3.4)	0.43	1.1 (0.4–2.7)	0.91
Galectin-3 (per ng/ml)	1.01 (0.97–1.05)	0.73	–	–	–		–	–
High Galectin-3 (≥2.6 ng/ml)	1.6 (0.7–3.6)	0.26	–	–	–		–	–
HbA1c > 70 mmol/mol (> 8.6%)	2.6 (1.3–5.1)	0.004	1.3 (0.6–3.0)	0.47	1.3 (0.6–3.0)	0.47	1.3 (0.6–3.0)	0.48
HDL-cholesterol	0.4 (0.2–1.0)	0.056	1.2 (0.4–3.5)	0.75	1.2 (0.4–3.5)	0.75	1.4 (0.4–4.2)	0.59
Continuous subcutaneous insulin infusion	0.2 (0.03–1.5)	0.12	–	–	–	–	–	–
Cardiovascular complications	2.5 (0.6–10.1)	0.19	–	–	–	–	–	–

a, b, c *Multiple logistic regression analyses (Backward: Wald); N = ^a, b, c^278; Nagelkerke R Square ^a, b^0.227/^c^0.258; Hosmer and Lemeshow ^a^0.148/^b^0.163/^c^0.475. In model 1, anxiety and abdominal obesity are included as separate variables. In model 2, combinations of anxiety and abdominal obesity are included. In model 3, high Gal3BP (≥ 3.3 μg/ml) is replaced by continuous Gal3BP. For missing values, see Tables 1, 2*.

The TAS-20 subscale DIF was correlated with Gal3BP (Pearson Correlation coefficient 0.136, *p* = 0.022), but not DDF (*p* = 0.55) or EOT (*p* = 0.66).

The ROC analyses showed that the area under the curve (lower bound-upper bound) was for alexithymia and Gal3BP 0.68 (0.59–0.77) (*p* < 0.001). For the chosen cut-off value, Gal3BP ≥3.3 μg/ml, the specificity was 0.85 and the sensitivity was 0.36. The area under the curve for depression and Gal3BP was 0.61 (0.50–0.71) (*p* = 0.054).

When alexithymia, depression and anxiety were tried against Gal3BP (Lg10), the analyses showed that alexithymia was associated with Gal3BP (Lg10) (unstandardized B coefficient 0.122, *p* < 0.001), which was neither the case for depression nor for anxiety (*p* = 0.89 and 0.94, respectively).

In bivariate correlations analyses, the Pearson Correlation coefficients were for Gal3BP and alexithymia 0.330 (*p* < 0.001), and for Gal3BP and depression 0.125 (*p* = 0.035). When partial correlation analysis was performed, controlling for alexithymia, the correlation coefficient for Gal3BP and depression was 0.044, (*p* = 0.46).

Collinearity analyses were calculated with alexithymia as dependent variable, and the included independent variables were age, diabetes duration, sex, depression, anxiety, abdominal obesity, Gal3BP, sCD163, HbA1c, and HDL. The results of the collinearity analyses showed that the collinearity tolerance coefficients were all ≥ 0.67 and the VIF coefficients were all ≤ 1.49.

Associations with high HbA1c (>70 mmol/mol) are presented in Table 4. Abdominal obesity (AOR 4.0, *p* < 0.001), high Gal3BP levels (AOR 2.8, *p* = 0.002), and depression (AOR 2.9, *p* = 0.014) were independently associated with high HbA1c.

**Table 4:** Associations with high levels of HbA1c.

	**HbA1** **>70 mmol/mol (>8.6%)**			
	**COR (95% CI)**	***P*-value**	**AOR (95% CI)**	***P*-value^a^**
Age (per year)	0.99 (0.97–1.01)	0.27	0.98 (0.95–1.00)	0.051
Diabetes duration (per year)	0.99 (0.97–1.02)	0.65	–	–
Sex (women)	1.5 (0.9–2.4)	0.16	0.8 (0.4–1.5)	0.49
Alexithymia	2.6 (1.3–5.1)	0.004	1.2 (0.5–2.7)	0.62
Depression	2.6 (1.2–5.6)	0.015	2.9 (1.2–6.9)	0.014
Anxiety	1.2 (0.7–2.0)	0.52	–	–
Abdominal obesity	3.3 (1.7–6.2)	<0.001	4.0 (2.0–8.1)	<0.001
High Gal3BP (≥ 3.3 μg/ml)	3.0 (1.7–5.5)	<0.001	2.8 (1.5–5.4)	0.002
High sCD163 (≥0.6 μg/ml)	1.7 (0.9–3.2)	0.081	1.3 (0.6–2.6)	0.46
High Galectin-3 (≥2.6 ng/ml)	0.6 (0.2–1.3)	0.17	–	–
HDL-cholesterol	0.5 (0.2–1.0)	0.040	0.8 (0.4–2.0)	0.69
Continuous subcutaneous insulin infusion	0.7 (0.3–1.9)	0.53	–	–

a *Multiple logistic regression analyses (Backward: Wald); N = 278; Nagelkerke R Square 0.166*.

*Hosmer and Lemeshow 0.885. For missing values, see Tables 1, 2*.

Abdominal obesity and anxiety were associated [COR (CI) 2.4 (1.3–4.5), *p* = 0.006]. There were neither any association between abdominal obesity and depression [COR (CI) 1.3 (0.5–3.4), *p* = 0.59] nor between abdominal obesity and high Gal3BP [COR (CI) (1.1 (0.5–2.4), *p* = 0.77].

Median (q_1_, q_3_) (μg/ml) Gal3BP was 3.1 (2.5, 4.0) for 10 persons with cardiovascular complications, and 2.3 (1.8, 3.0) (*p* = 0.050) for 275 patients without cardiovascular complications. Associations with cardiovascular complications were in univariate analyses significant for depression [COR (CI) 6.6 (1.7–24.8), *p* = 0.005] and for Gal3BP (per μg/ml) [COR (CI) 1.5 (1.0–2.2), *p* = 0.039], but not for alexithymia [COR (CI) 2.5 (0.6–10.1), *p* = 0.19]. In multivariate analyses depression remained significantly associated with cardiovascular complications [AOR (CI) 5.4 (1.4–21.1), *p* = 0.016], but not Gal3BP [AOR (CI) 1.4 (0.9–2.1), *p* = 0.11].

## Discussion

### Main Findings

**Figure 1:**
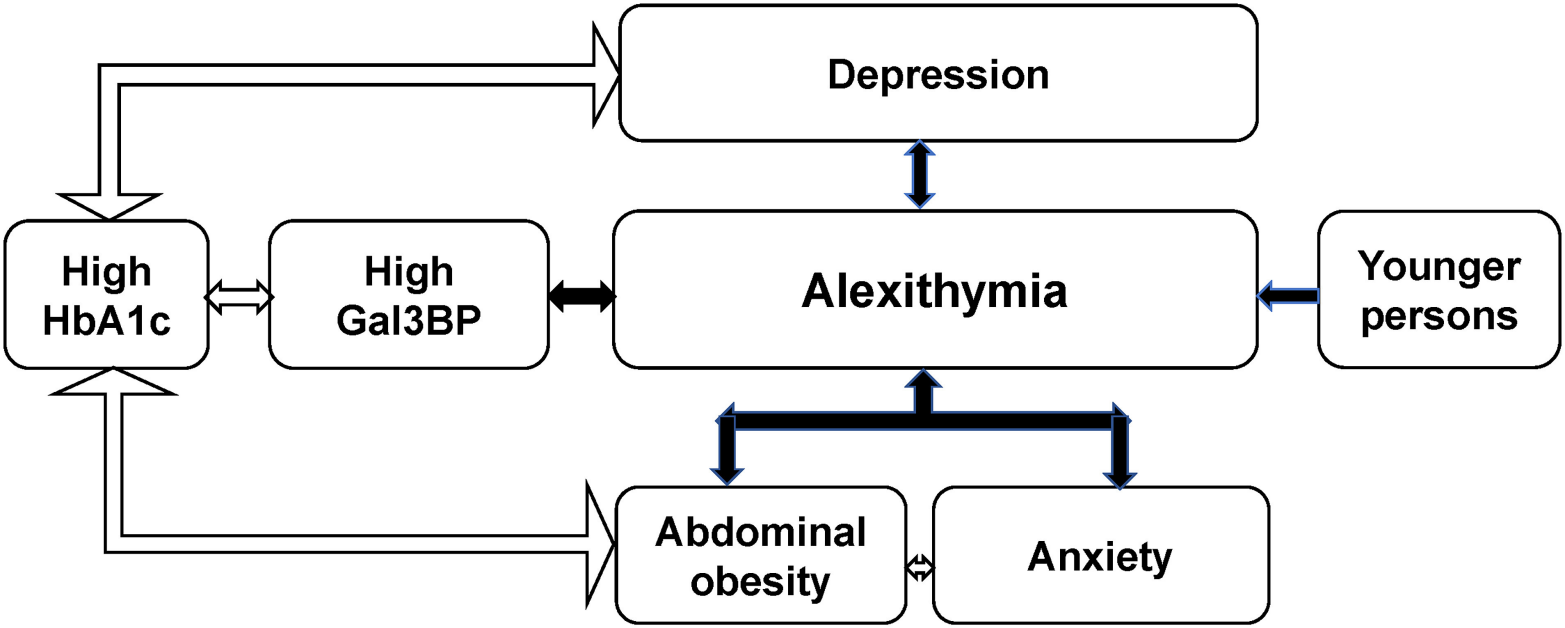
Graphic illustration of the demonstrated significant associations.

The main findings of this study of 292 adult patients with T1D were that high Gal3BP levels, depression and younger age were independently associated with alexithymia. Abdominal obesity, anxiety and high HbA1c levels (> 70 mmol/mol) were associated with alexithymia in univariate analyses. Abdominal obesity was only in combination with anxiety associated with alexithymia in multivariate analyses. Furthermore, high HbA1c levels were not independently associated with alexithymia though high HbA1c levels were associated with three different variables, abdominal obesity, high Gal3BP levels and depression, see Figure 1. The main alexithymia subfactor was difficulty identifying feelings (DIF), which was correlated with Gal3BP.

### Alexithymia and Immunity

Gal3BP, which previously was linked to metabolic disturbances, cardiovascular disease and mortality (21, 22, 27), was the only immuno-inflammatory variable independently associated with alexithymia. Gal3BP is important as it has the potential to induce pro-inflammatory cytokines (21, 26). Higher levels of Gal3B has been demonstrated in people with diabetes mellitus compared to people without diabetes (21). In these T1D patients, there was an association between high Gal3BP levels and impaired glycemic control expressed as high HbA1c levels. In patients with previous myocardial infarction, high Gal3BP levels and diabetes mellitus were independently associated with increased risk for the development of angina pectoris or reinfarction (22). Gal3BP has been suggested as a novel important biomarker for cardiovascular risk assessment (22). To our knowledge, an association between alexithymia and Gal3BP has not previously been demonstrated.

### Alexithymia and Depression

Depression is a clear risk factor for cardiovascular disease and all-cause mortality (38). The patients with alexithymia in this study had a very high prevalence of depression, which is in accordance with previous research (9, 14–16). Alexithymia has been suggested as a risk factor for depression even though alexithymic features seem to increase during depressive episodes (55, 56). Several immuno-inflammatory disturbances have been demonstrated in depressive states such as increased levels of galectin-3 and decreased soluble TWEAK (31, 32, 41, 43). Galectin-3 not only serves as a biomarker, but is also a contributor to cardiovascular disease and mortality (22, 24, 25).

### Alexithymia and Metabolic Disturbances

Alexithymia has previously been linked to obesity and binge eating disorders (11). Obesity is a major risk factor for cardiovascular disease (13, 21, 34) and there is some evidence that anxiety contributes to cardiovascular disease (39). Anxiety and abdominal obesity were closely linked in this study, and these two factors combined were associated with alexithymia. One possible mechanism is that patients with alexithymia cannot discriminate between the unease caused by anxiety and the unease caused by hunger. Another possibility is that people with alexithymia cannot distinguish between symptoms of hypoglycaemia and anxiety. Difficulty in distinguishing between bodily sensations due to emotional arousal and due to other causes is a main characteristic of alexithymia (2). In both cases, the consequences of this particular alexithymia feature may be increased food intake with subsequent weight gain. Obesity might also cause anxiety, as patients with diabetes are regularly informed about the deleterious impact obesity may have on their health (34, 49).

The patients with and without alexithymia did neither differ by levels of blood pressure or s-lipids, nor by the prevalence of antihypertensive medication or lipid-lowering drugs usage, which differ from previous research (13).

### Alexithymia and Life Style

Smoking habits and levels of physical activity didn't differ between patients with and without alexithymia.

### Potential Explanatory Mechanisms

As this study is a cross-sectional study, we cannot determine any causality. We will, however, discuss potential explanatory mechanisms. There is evidence from previous research that parts of the innate immune system which during the evolution were developed to fight pathogens, might be activated by environmental psychosocial stress or by the anticipation of perceived danger (43, 44). Persons with alexithymia may be inclined to anticipate danger, particularly if they during childhood were exposed to physical abuse, emotional neglect or other types of trauma, which are risk factors for alexithymia (5). According to previous research alexithymia is characterized by inflexible emotional dysregulation with dysfunctional emotional reactivity and prolonged emotional states (4), which potentially could trigger prolonged immune reactions in case of perceived or anticipated danger. The immune system acts in a coordinated way with the sympathetic nervous system and the HPA-axis (2, 3, 43). The increased baseline sympathetic activity or the disturbances of the HPA axis observed in alexithymia (2, 3), may trigger the immune system with increased levels of Gal3BP. The increased Gal3BP levels are deleterious for patients with T1D as Gal3BP induces a number of pro-inflammatory cytokines (21). Increased levels of Gal3BP contribute to metabolic disturbances (27), and increased Gal3BP levels are risk factors for cardiovascular disease and all-cause mortality (21, 22).

Blood glucose levels also increase by activation of the stress system (57), which may contribute to the demonstrated increased HbA1c levels.

### Future Research

In future research it would be of interest to explore the impact of alexithymia, Gal3BP and depression on cardiovascular complications in a longitudinal study. Another subject for research would be to explore whether therapies aiming at increased emotional awareness could ameliorate alexithymia features (51). At a molecular level it would be of interest to further explore stress, alexithymia, depression, sterile inflammation and danger-associated molecular patterns (DAMP) (44).

### Strengths and Limitations of the Study

Strengths of our study are that patients with severe comorbidities such as cancer, severe autoimmune disorders, hepatic failure, end-stage renal disease, psychotic and bipolar disorders were excluded as these disorders, or medication for these disorders, may have impact on the immune system (26, 27). We controlled for relevant variables previously linked to cardiovascular disease and mortality. The logistic regression models were elaborated for the associations, and calibrated and validated for goodness of fit with the data variables. The included variables were checked for multicollinearity, and no significant evidence of multicollinearity was demonstrated. ROC analyses were performed and the area under the curve was only significant for Gal3BP and alexithymia, not for depression. Multiple linear regression analysis was performed and alexithymia was associated with log transformed Gal3BP, which was not the case for depression. Altogether, these analyses support the result of the multiple logistic regression analyses, Gal3BP and depression were independently associated with alexithymia. Finally, precise ELISA techniques were used and showed low intra-assay coefficients of variation for Gal3BP, sCD163 and galectin-3 (31, 47, 48).

Cross-sectional studies have limitations as causality cannot be confirmed. Only a limited number of patients with cardiovascular disease participated in the study, so an association between Gal3BP and cardiovascular complications could neither be confirmed nor excluded. The *P*-value was quite low, which could indicate a type-2 error. Depression, anxiety and alexithymia were only assessed by self-report instruments, not by a structured interview. TAS-20 is, however, extensively used in research, and evidence collected for 26 years supports that the scale adequately measures the alexithymia construct (3, 8, 9, 11, 14, 17, 18, 52). HADS has also shown high validity for assessing symptoms of depression and anxiety both at an individual and a collective level (53).

## Conclusions

Gal3BP, depression and younger age were independently associated with alexithymia. The patients with alexithymia also had higher levels of HbA1c, and higher prevalence of abdominal obesity and anxiety, but these factors were not independently linked to alexithymia. The findings of this study contribute to the understanding of the complex processes which may contribute to the development of cardiovascular disease in patients with T1D and alexithymia.

## Data Availability Statement

The raw data supporting the conclusions of this article will be made available by the authors, without undue reservation.

## Ethics Statement

The studies involving human participants were reviewed and approved by Regional Ethical Review Board of Linköping University, Linköping, Sweden (Registration no. M120-07, T89-08). The patients/participants provided their written informed consent to participate in this study.

## Author Contributions

RS, JD, MH, and EM participated as investigators and reviewed, edited, and approved the final version of the manuscript. RS contributed with his knowledge of psychology and initiated the research group's interest in the health implications of alexithymia. JD and MH contributed with their knowledge of immunology, performed the analyses of the immuno-inflammatory variables, and they take the full responsibility for these analyses. EM integrated the knowledge of psychology, clinical medicine and immunology, performed the statistical analysis, is the guarantor of this work and, as such, had full access to all the data in the study, and takes responsibility for the integrity of the data and the accuracy of the data analysis.

## Conflict of Interest

The authors declare that the research was conducted in the absence of any commercial or financial relationships that could be construed as a potential conflict of interest.
